# Insights into Drug Donation Practices and Public Perceptions in Saudi Arabia

**DOI:** 10.3390/healthcare12192001

**Published:** 2024-10-07

**Authors:** Ibrahim Alredaini, Nada Fayez Alshehri, Khadijah Jameel Muzayen, Renad Alalwani, Ghosoon Bafaraj, Abdullah S. Alshammari, Adnan S. Alharbi, Hazim M. AlHuzaym, Mahmoud Elrggal, Abdulmalik S. Alotaibi, Nasser M. Alorfi, Mohammed Alrashed, Abdullah A. Alhifany, Mohammed A. Alnuhait

**Affiliations:** 1 Clinical Research Department, King Faisal Specialist Hospital and Research Centre, Riyadh 12713, Saudi Arabia; ialredaini@kfshrc.edu.sa; 2 Pharmaceutical Practices Department, College of Pharmacy, Umm Al-Qura University, Makkah 24382, Saudi Arabia; s441000064@st.uqu.edu.sa (N.F.A.); s442012950@st.uqu.edu.sa (K.J.M.); s441012123@st.uqu.edu.sa (R.A.); ghosoon.m@hotmail.com (G.B.); asshammari@uqu.edu.sa (A.S.A.); assharbi@uqu.edu.sa (A.S.A.); assalotaibi@uqu.edu.sa (A.S.A.); aahifany@uqu.edu.sa (A.A.A.); 3 Inpatient Pharmacy Department, Prince Sultan Military Medical City (PSMMC), Riyadh 12233, Saudi Arabia; halhuzaym@psmmc.med.sa; 4 Pharmacology and Toxicology Department, Faculty of Medicine, Al Qunfudah, Umm Al-Qura University, Makkah 24382, Saudi Arabia; merggal@uqu.edu.sa; 5 Pharmacology and Toxicology Department, College of Pharmacy, Umm Al-Qura University, Makkah 24382, Saudi Arabia; nmorfi@uqu.edu.sa; 6 King Abdullah International Medical Research Center, Riyadh 11481, Saudi Arabia; alrashidm@ksau-hs.edu.sa; 7 Department of Pharmacy Practice, College of Pharmacy, King Saud bin Abdulaziz University for Health Sciences, Riyadh 11481, Saudi Arabia; 8 Pharmaceutical Care Department, King Abdulaziz Medical City, National Guard Health Affairs, Riyadh 11426, Saudi Arabia

**Keywords:** pharmacy, drug, Saudi Arabia, donations, medication

## Abstract

Background: Effective medication management, disposal, and donation are vital for public health and environmental sustainability. Improper handling of medications can lead to drug misuse, accidental poisoning, and environmental damage. This study examines current practices and challenges in Saudi Arabia, identifying opportunities for improvement. Method: A cross-sectional survey conducted in October and November 2023 targeted the general population in Saudi Arabia. This study employed convenience sampling to explore medication usage, storage, disposal practices, and awareness of donation procedures. Data were analyzed through both descriptive and inferential statistical methods. Results: This study involved 430 respondents. Of these, 73.0% held a university degree, yet 66.3% were unaware of drug donation programs, and 84.2% lacked knowledge about proper medication disposal. Despite this, 71.4% believed that drug donation programs positively impact healthcare, and 87.9% saw them as reducing drug waste and environmental pollution. However, 48.1% expressed concerns about the potential misuse of donated medicines. Awareness and knowledge were significantly higher among participants aged 30 and above. Conclusions: The findings highlight the need for enhanced public awareness, clear medication disposal guidelines, and ethically governed donation practices in Saudi Arabia. These measures can improve healthcare outcomes, protect the environment, and support global health and sustainability goals.

## 1. Introduction

The improper disposal of unused medicines poses a significant risk to public health [1]. Accumulating these medicines in homes increases the risk of accidental poisoning or deliberate misuse, potentially leading to suicide [1]. Therefore, it is essential to both reduce the generation of unused medicines and ensure their safe and appropriate disposal. This public health issue demands serious attention and responsible action [2]. Drug donation represents a viable method for the disposal of medications, effectively redirecting unused drugs to where they are needed most [3]. Drug donation practices are used for a variety of reasons, ranging from providing emergency relief to sustaining long-term medicinal supplies or even for recycling drugs. A common scenario is the donation of leftover medications nearing their expiry date, which often occurs when clinics purchase more than they need. Although the origins and intentions behind drug donations may differ, they are all guided by a set of universal basic guidelines, ensuring that these donations are handled responsibly and effectively. This approach helps to maximize the benefits of drug donations while minimizing potential risks [4]. The accumulation of unused medicines in patients’ homes has prompted charitable organizations to step in, collecting and redistributing these medications before they expire as a way to reduce waste and ensure their proper use [5,6]. Drug donations, often provided free of charge, are supplied to various countries by individuals, organizations, and non-governmental organizations (NGOs) [2]. In any medical practice, improperly managed donations can create more burdens than benefits for the intended recipient governments. The World Health Organization (WHO) published its first guidelines for drug donations in 1996, with contributions from various parties, including United Nations agencies, international humanitarian relief organizations, and others [7]. In 2010, the third and final edition of the Medical Donations Guidelines was issued, featuring twelve best-practice recommendations for both donor and recipient countries. These recommendations specify that donations should meet the recipient country’s specific needs, align with its disease patterns, and be in agreed quantities. Donated medicines should be approved for use in the recipient country, listed on its national list of essential medicines or standard treatment guidelines, and similar to those commonly used there. They must originate from a quality-assured source and meet quality standards [8]. In Saudi Arabia, the situation of drug donations is unique and warrants specific attention since the awareness and perception of community practices regarding drug donations represent an area lacking regulation and specific policy guidance. This study aims to thoroughly survey and assess the current practices and community perceptions of drug donations within Saudi Arabia, evaluating their alignment with international norms and identifying unique local challenges. By highlighting these insights, this research intends to illuminate the path toward establishing effective management and regulatory frameworks for drug donations, thereby enhancing public health outcomes and ensuring environmental safety in Saudi Arabia.

## 2. Method

### 2.1. Study Design

A cross-sectional survey design was employed to evaluate the attitudes and perceptions of the Saudi population toward drug donation. Conducted electronically and distributed via social media throughout October and November 2023, the survey aimed to reach a diverse demographic across Saudi Arabia. We hypothesized that there might be a general lack of significant interest and willingness to participate in drug donation activities among the Saudi population. This assumption was based on a few key factors: limited public awareness about drug donation programs, cultural attitudes toward medication sharing, and potential concerns regarding the safety and efficacy of donated drugs. Additionally, we anticipated that knowledge gaps and a lack of institutional support could further discourage participation in drug donation. For our study, we estimated that about 50% of the population would be aware of the concept of drug donation. To determine the appropriate sample size, we used a 5% margin of error and a 95% confidence interval, considering Saudi Arabia’s population of around 32 million [9] (General Authority for Statistics, no date). This calculation led us to aim for a sample size of 385 participants. We employed convenience sampling, targeting Saudi citizens aged 18 and older, to gather a broad understanding of public attitudes toward drug donation and provide insights that could inform future initiatives. All participants aged 18 and above, regardless of their prior awareness of drug donation programs, were included to capture both awareness levels and general public perceptions, including potential benefits and barriers, after being introduced to the concept in the survey. Participants were informed about the study’s purpose and assured of their anonymity and data confidentiality. On average, completing the survey took around 10 min. This study sought to illuminate current practices related to medication donation in Saudi Arabia and identify areas for potential improvement. This study received ethical approval from Umm Al-Qura University, approval number HAPO-02-K-012-2023-10-1824.

### 2.2. Questionnaires and Data Collection

The survey was conducted using an online platform, with participants accessing it through a provided link via Google Forms. The development and validation of the questionnaire was grounded in an extensive review of relevant literature and expert consultations in drug donation and pharmacy [8,10]. The validation of our survey instrument was thorough, encompassing several stages. We undertook pilot testing and validity assessments to refine its clarity and relevance. The reliability analysis, which confirmed the internal consistency, was a crucial part of this process. During the initial pilot phase, feedback from 10 participants was instrumental in enhancing the survey’s design and content. The questionnaire was available in both Arabic and English to ensure accessibility and inclusivity for a diverse range of participants. The questionnaire for our study consisted of 44 items categorized into seven distinct sections. Section A focused on gathering demographic information of the participants. Section B explored the awareness of drug donation programs, assessing the participants’ knowledge and understanding of these programs. In Section C, types of medication donations were examined, examining various forms and categories of donations. Section D explored perceptions of drug donation programs, seeking to understand how participants viewed these initiatives. Factors influencing participation were the focus of Section E, which aimed to identify what drives or hinders individuals from participating in these programs. Section F addressed the ethical considerations and contributions of drug donations to community service, exploring the moral implications and community impact of drug donations. Finally, Section H evaluated the willingness to participate in the drug donation program, assessing the participants’ readiness to be involved in such initiatives. Each section was designed to provide a thorough understanding of the various aspects of drug donation practices.

### 2.3. Statistical Analysis

This study involved rigorous data handling before statistical analysis. The data were cleaned to correct any inconsistencies, missing values, or outliers. The data were then anonymized, coded, and categorized for efficient analysis, ensuring participant confidentiality. Categorical variables are presented as frequencies and percentages, whereas continuous variables are presented as means and standard deviations. Modified Poisson regression analyses using Sandwich estimation were used to model the effect of demographic information on different aspects of drug donation programs, including awareness about their existence, knowledge about their details, familiarity with regulations surrounding them, perceptions of their importance, perceptions of their positive impact, and willingness to participate in those programs. The use of modified Poisson models was decided in light of the high prevalences seen for the outcomes in the current data. A significance level of 0.05 was used in all analyses. All analyses were conducted using R software version 4.2.2.

## 3. Result

The study included 430 participants whose demographic information is presented in Table 1. The majority were female (73.0%), and the age distribution was diverse, with a mean age of 32.5 (±11.2) years. The age breakdown indicated a significant proportion of individuals aged 20 to less than 30 years (41.6%). The study predominantly included Saudi participants (95.1%), reflecting the regional demographic composition. Marital status was evenly distributed, with 47.2% married and 46.5% single participants. In terms of education, 73.0% held a university degree. Employment status varied, with 42.3% employed and 29.3% being students. The monthly household income distribution was diverse, with 41.9% falling in the 6000 to 15,000 SAR range. Geographically, participants were distributed across provinces, with Mecca (41.4%), Madinah (27.7%), and Riyadh (12.8%) being the most represented.

Table 2 presents an overview of the respondents’ awareness of drug donation programs and the necessity of such initiatives in Saudi Arabia. A significant proportion of participants (66.3%) reported not having heard about drug donation programs, whereas 23.0% affirmed awareness. Among those aware, social media emerged as the primary source of information (12.1%), followed by family or friends (5.6%) and healthcare professionals (4.7%). Awareness of drug donation centers or collection points was low, with only 12.1% acknowledging their existence. Regarding knowledge levels, the majority indicated limited familiarity with program details (84.2%).

The responses to questions about the awareness and perceived necessity of different types of medication donations reveal valuable insights and are presented in Table 3. A substantial proportion of participants were aware that various medication forms could be donated, with a majority recognizing the suitability of oral medications (67.2%), topical medications (46.3%), and inhalers or respiratory medications (42.3%) for donation. However, there was a notable lack of awareness regarding donation possibilities, as indicated by the 9.5% who responded as “Not aware”. When expressing preferences on the types of medications needed in Saudi Arabia, respondents predominantly favor oral medications (64.0%), inhalers or respiratory medications (55.1%), and injectable medications such as insulin (44.9%).

The perceptions of drug donation programs among the respondents provide valuable insights into the potential impact and challenges associated with such initiatives in Saudi Arabia, as shown in Table 4. A significant majority (83.7%) acknowledged the importance of drug donation programs, underscoring their perceived value in contributing to healthcare. Participants foresaw various benefits from these programs, with the most emphasized being the facilitation of the donation process (69.8%), increased awareness of the culture of drug donation (76.0%), and the reduction in medication waste (69.1%). Concerns about the safe use and potential misuse of donated medication emerged as a prominent barrier, highlighted by 69.1% of respondents. Despite some concerns, a majority (71.4%) believe that drug donation programs positively impact healthcare in Saudi Arabia. Notably, when asked about improvements that can be made to enhance the impact and effectiveness of drug donation programs in Saudi Arabia, responses centered on the importance of information, awareness, and marketing (67.0%).

The factors influencing participation in drug donation programs among the respondents revealed key considerations that could impact their willingness to engage in such initiatives (Table 5). A substantial majority (87.4%) expressed the motivation to participate when they knew it would benefit those in need. Additionally, participants emphasized the importance of a simplified donation process (45.6%), trust in the program’s organization (46.5%), community support and awareness (57.2%), and clear information about donation locations and procedures (43.0%). Notably, a significant portion believed that drug donation can reduce drug waste (87.9%) and environmental pollution (75.6%). Respondents also expressed concern about the lack of drug donation programs in Saudi Arabia (26.7%) and the need for regulations governing pharmaceutical donations in Arab countries (86.3%). The large number of “neutral” responses (59.1%) about the existence of drug donation programs in Saudi Arabia indicates that many participants were uncertain or lacked enough knowledge to give a clear answer. This highlights the general uncertainty, even among those who had not heard of the programs.

The responses to questions about the ethical contributions of drug donation programs revealed a positive perception of their societal impact among the participants (Table 6). An overwhelming majority (92.3%) agreed that drug donation programs contribute to community service. Furthermore, a significant proportion (69.3%) believed that these programs could play a role in reducing unethical trade in medicines. Regarding knowledge of legislation and regulations related to drug donation, a substantial number of participants expressed varying levels of awareness, with a notable percentage acknowledging limited knowledge (77%). Interestingly, a substantial portion (48.1%) expressed concerns about the potential misuse of donated medicines for fraudulent purposes.

The findings from the responses to questions about the willingness to participate in drug donation programs provided valuable insights into the factors influencing individuals’ decisions. Most respondents (89.3%) reported no previous experience in drug donation, whereas 10.7% indicated having participated before. Of those with experience, 31 respondents chose to describe their experience. Among them, only four respondents reported that they donated through a drug donation program, while the others described personal donations. Notably, most participants (83.7%) expressed a willingness to participate if they knew that the donated medication would directly benefit individuals in their community. When asked about factors influencing their decision to participate, participants highlighted the significance of clear guidelines on acceptable donations (76.7%), the assurance of proper disposal of expired medications (66.0%), and the convenience of donation drop-off locations (67.0%). Interestingly, a sizeable proportion (29.5%) noted the importance of incentives for donors, such as tax benefits or acknowledgments.

### Effects of Demographic Characteristics on the Awareness, Perceptions, and Willingness to Participate in Drug Donation Programs

The results of the modified Poisson regression analyses reveal significant associations between demographic factors and the awareness of drug donation programs in terms of existence, program details, and familiarity with regulations. Participants aged 30 to less than 40, 40 to less than 50, and those aged 50 and above demonstrated significantly higher awareness about the existence of drug donation programs compared with the reference group (20 to less than 30), with PRs of 3.03 (95% CI: 1.31, 7.04, *p* = 0.010), 3.32 (95% CI: 1.41, 7.82, *p* = 0.006), and 3.52 (95% CI: 1.12, 11.06, *p* = 0.032), respectively. Participants aged 40 to less than 50 exhibited increased knowledge about program details, showing a PR of 2.93 (95% CI: 1.06, 8.12, *p* = 0.039). Moreover, individuals with a monthly household income of more than SAR 50,000 were significantly more aware of the existence of drug donation programs (PR = 5.12, 95% CI: 1.05, 24.89, *p* = 0.043). Notably, other demographic factors, including gender, nationality, marital status, educational level, employment status, and province, were not significantly associated with awareness, knowledge, or familiarity with drug donation programs.

The modified Poisson regression analyses investigated the impact of respondents’ demographic characteristics on their perception of the importance of drug donation programs, their positive impact, and willingness to participate in such programs. Noteworthy associations include the influence of age, employment status, and monthly household income. Participants aged 40 to 50 years were more likely to perceive the importance of drug donation programs (PR = 1.19, 95% CI: 1.00, 1.42, *p* = 0.050) but less likely to perceive their current positive impact (PR = 0.77, 95% CI: 0.59, 0.99, *p* = 0.044) compared with the reference group (20 to less than 30). Single individuals were more likely to perceive the importance of drug donation programs (PR = 1.13, 95% CI: 1.01, 1.26, *p* = 0.032) than their married counterparts. Students were less likely to perceive the current positive impact of drug donation programs (PR = 0.78, 95% CI: 0.65, 0.94, *p* = 0.007), and unemployed individuals were less willing to participate (PR = 0.85, 95% CI: 0.73, 0.99, *p* = 0.036) compared with employed individuals. Moreover, participants with a monthly household income of more than SAR 15,000 to 30,000 were more likely to perceive the current positive impact of drug donation programs (PR = 1.18, 95% CI: 1.03, 1.35, *p* = 0.020) than those with a monthly household income of more than SAR 6000 to 15,000. No significant associations were found for the other demographic factors.

## 4. Discussion

In this study, we explored the awareness and perceptions surrounding drug donation programs among participants. A significant 66.3% of respondents were not aware of such programs, with social media emerging as a key information source for those who were informed. This study underscores the pervasive issue of inadequate knowledge regarding proper medication disposal and donation practices, as highlighted by Althagafi et al. (2022), who noted that 12.9% of individuals donate their unused medications, thereby contributing to a culture of medication redistribution within communities [11].

Only 12.1% of participants knew about drug donation centers, despite a substantial majority (90.2%) recognizing the urgent need for increased public awareness. The types of medications deemed suitable for donation were predominantly oral, reflecting concerns about medication integrity, potential abuse, and regulatory challenges, similar to other studies [12,13].

This study also revealed strong support (83.7%) for drug donation programs as a means to alleviate health disparities and provide emergency assistance. Yet, 69.1% of participants believed these programs could minimize medication waste. Notwithstanding the evident support for drug donation initiatives, apprehensions regarding privacy, legal issues, and safety were prominent, paralleling a Dutch study where 61.2% of patients expressed willingness to use returned medication, contingent on quality assurance [14]. Another study uncovered disposal practices among respondents, with a significant 73% admitting to discarding medications in the trash. Conversely, 14% reported returning unused medications to pharmacies, while only 3% of participants engaged in the practice of donating their medications to friends or charity centers [15]. Also, another study highlights the effectiveness of permanent drug donation boxes in safely removing controlled substances from communities, reducing potential misuse. Ensuring convenient access to these disposal options for both rural and urban populations is crucial for improving public safety and health [16].

Most people viewed the ethics of drug donation programs positively, though some were concerned about the potential for misuse or fraud. The study also examined drug donation policies, finding that while they generally follow WHO guidelines, there is still a lack of clear policies for both donors and recipients [8]. Demographic variables such as age and income level markedly influenced awareness and perceptions, with middle-aged and higher-income groups showing greater program awareness. This aligns with another study that emphasized the necessity for improved public education on drug donation and disposal, advocating for the use of social networks to expand awareness [11].

To enhance the safety and utilization of unused medicines, it is imperative to advocate for the establishment of approved policies and regulations governing drug donations in Saudi Arabia. Such measures will ensure the safe and effective redistribution of medications, thereby regulating the practice and maximizing its benefits to society. This study is not without its limitations. A limitation of this study is the use of convenience sampling, which may restrict the generalizability of the findings due to potential selection bias, despite efforts to achieve broad geographic distribution across different regions of Saudi Arabia. Also, another limitation of this study is that online data collection may have led to a lower number of older adult participants, who are regular medication users. The reliance on self-reported data may introduce bias. Additionally, the cross-sectional design limits the ability to ascertain causality or track changes over time. Future research should aim to diversify the participant pool and employ longitudinal designs to observe trends. There is also a need for intervention studies to test the effectiveness of different educational strategies on enhancing awareness and perceptions regarding drug donation programs.

## 5. Conclusions

This study revealed that most people in Saudi Arabia are unaware of drug donation programs, though many recognize their potential benefits for healthcare. Concerns were raised about safety and unclear procedures, but there is a strong interest in participating if these issues are addressed. Increasing awareness, simplifying the process, and establishing clearer regulations could encourage more people to get involved and help reduce medication waste.

## Figures and Tables

**Table 1:** Demographic information.

Characteristics	No. (*n* = 430)	%
**Gender**		
Female	314	73
Male	116	27
**Age (years)**		
Less than 20	24	5.6
20 to less than 30	179	41.6
30 to less than 40	101	23.5
40 to less than 50	90	20.9
50+	36	8.4
**Nationality**		
Saudi	409	95.1
Non-Saudi	21	4.9
**Marital status**		
Married	203	47.2
Divorced	11	2.6
Single	200	46.5
Widow	6	1.4
Prefer not to answer	10	2.3
**Educational level**		
Postgraduate	28	6.5
University	314	73
High school or diploma	77	17.9
Intermediate or less	11	2.6
**Employment Status**		
Employed	182	42.3
Self-employed	10	2.3
Unemployed	90	20.9
Retired	22	5.1
Student	126	29.3
**Monthly household income**		
Less than SAR 6000	67	15.6
SAR 6000 to 15,000	180	41.9
More than SAR 15,000 to 30,000	88	20.5
More than SAR 30,000 to 50,000	10	2.3
More SAR than 50,000	9	2.1
Prefer not to answer	76	17.7
**Province**		
Mecca	178	41.4
Madinah	119	27.7
Riyadh	55	12.8
Northern	22	5.1
Qassim	21	4.9
Eastern	16	3.7
Southern	12	2.8
Prefer not to answer	7	1.6

**Table 2:** Distribution of answers to questions about awareness of drug donation programs.

Questions	No. (*n* = 430)	%
**Have you ever heard of drug donation programs?**		
No	285	66.3
Not sure	46	10.7
Yes	99	23
**If yes, how did you first learn about them?**		
Social media	52	12.1
Family or friends	24	5.6
HCP	20	4.7
Others	3	0.7
Did not answer with “Yes”	331	77
**Are you aware of any drug donation centers or collection points in your vicinity?**		
No	378	87.9
Yes	52	12.1
**How would you rate your knowledge level regarding the details of these programs?**		
Unknowledgeable	186	43.3
Not very knowledgeable	176	40.9
Somewhat knowledgeable	54	12.6
Very knowledgeable	14	3.3
**Are you familiar with the regulations and guidelines surrounding pharmaceutical donation in your country?**		
No	350	81.4
Not sure	43	10
Yes	37	8.6
**How widespread do you believe drug donation programs are in Saudi Arabia?**		
Not very widespread	196	45.6
Not widespread at all	126	29.3
Somewhat widespread	81	18.8
Very widespread	25	5.8
Missing	2	0.5
**Have you or anyone you know ever benefited from donated medications?**		
No, neither I nor anyone I know have benefited	326	75.8
Yes, I have benefited	31	7.2
Yes, someone I know has benefited	73	17
**Do you think there is a need for increased public awareness regarding the types of medications that can be donated?**		
Yes, definitely	388	90.2
Yes, to some extent	34	7.9
No not at all	8	1.9

**Table 3:** Distribution of answers to questions about the types of medication donations.

Questions	No. (*n* = 430)	%
**Are you aware that different types of medications can be donated?**		
Oral medications (tablets, capsules)	289	67.2
Syrups or liquid medications	148	34.4
Inhalers or respiratory medications	182	42.3
Injectable medications (e.g., insulin)	108	25.1
Intravenous (IV) medications	48	11.2
Topical medications (creams, ointments)	199	46.3
Not aware	41	9.5
**In your opinion, which type of medication donations do you think are most needed in Saudi Arabia?**		
Oral medications (tablets, capsules)	275	64
Syrups or liquid medications	143	33.3
Inhalers or respiratory medications	237	55.1
Injectable medications (e.g., insulin)	193	44.9
Intravenous (IV) medications	88	20.5
Topical medications (creams, ointments)	152	35.3
No opinion	16	3.7

**Table 4:** Distribution of answers to questions about perceptions of drug donation programs.

Questions	No. (*n* = 430)	%
**How do you perceive the importance of drug donation programs in Saudi Arabia?**		
Important	360	83.7
Neutral	66	15.3
Unimportant	4	0.9
**What benefits do you expect from the drug donation program?**		
Facilitating the process of donating medicines	300	69.8
Increase awareness of the culture of drug donation	327	76
Ensure the safety of the medications	229	53.3
Reducing medication waste	297	69.1
**What potential barriers or concerns, if any, would prevent you from donating medication to a drug donation program?**		
Concerns about the safe use and potential misuse of the donated medication would deter me from donating	297	69.1
If I do not have enough information about where or how to donate, it would prevent me from donating	229	53.3
Worries about the privacy of my personal health information	86	20
If the process of donating medicines is complicated or takes a long time, this does not encourage me to donate	179	41.6
Proper storage	3	0.7
**Do you think drug donation programs positively impact healthcare in Saudi Arabia?**		
Agree	307	71.4
Disagree	20	4.7
Neutral	103	24
**What improvements can be made to enhance the impact and effectiveness of drug donation programs in Saudi Arabia?**		
Information, awareness, and marketing	288	67
No opinion	142	33

**Table 5:** Distribution of answers to questions about factors influencing participation in drug donation programs.

Questions	No. (*n* = 430)	%
**What factors motivate you to participate in a drug donation program?**		
Knowing that it benefits those in need	376	87.4
Simplified donation process	196	45.6
Trust in the program’s organization	200	46.5
Community support and awareness	246	57.2
Clear information about donation locations and procedures	185	43
**I believe that the drug donation program reduces drug waste by providing other means of using medications**		
Agree	378	87.9
Disagree	6	1.4
Neutral	46	10.7
**I believe that donating medicines reduces environmental pollution**		
Agree	325	75.6
Disagree	13	3
Neutral	92	21.4
**There is no drug donation programs in Saudi Arabia**		
Agree	115	26.7
Disagree	61	14.2
Neutral	254	59.1
**Pharmaceutical donations in Arab countries are not subject to regulations**		
Agree	68	15.8
Disagree	132	30.7
Neutral	230	53.5
**I believe that drug donation must be subject to laws for the risk of being exploited for personal interests.**		
Agree	371	86.3
Disagree	6	1.4
Neutral	53	12.3

**Table 6:** Distribution of answers to questions about the ethical contribution of drug donation programs.

Questions	No. (*n* = 430)	%
**Do you think the drug donation program contributes to community service?**		
Agree	397	92.3
Disagree	2	0.5
Neutral	31	7.2
**Do you feel that the drug donation program could reduce unethical trade in medicines?**		
Agree	298	69.3
Disagree	26	6
Neutral	106	24.7
**How aware are you of the legislation and regulations related to drug donation?**		
Unknowledgeable	150	34.9
Not very knowledgeable	181	42.1
Somewhat knowledgeable	62	14.4
Very knowledgeable	37	8.6
**Do you think that donating medicines may be used as a means of fraud?**		
Agree	207	48.1
Disagree	60	14
Neutral	163	37

## Data Availability

The data presented in this study are available on request from the corresponding author due to privacy and ethical restrictions.
